# Aromatase Inhibitors as Adjuvant Treatment for ER/PgR Positive Stage I Endometrial Carcinoma: A Retrospective Cohort Study

**DOI:** 10.3390/ijms21062227

**Published:** 2020-03-23

**Authors:** Laura Paleari, Mariangela Rutigliani, Giacomo Siri, Nicoletta Provinciali, Nicoletta Colombo, Andrea Decensi

**Affiliations:** 1 A.Li.Sa., Liguria Region Health Authority, 16121 Genoa, Italy; 2 Pathology Unit, Galliera Hospital, 16128 Genoa, Italy; mariangela.rutigliani@galliera.it; 3 Office of the Scientific Director, Galliera Hospital, 16128 Genoa, Italy; giacomo.siri@galliera.it; 4 Medical Oncology Unit, Galliera Hospital, 16128 Genoa, Italy; nicoletta.provinciali@galliera.it (N.P.); andrea.decensi@galliera.it (A.D.); 5 Gynecology Program, European Institute of Oncology, 20141 Milan, Italy; nicoletta.colombo@ieo.it; 6 School of Medicine and Surgery, University Milan Bicocca, 20126 Milan, Italy; 7 Barts School of Medicine, Queen Mary University of London, London E1 4NS, UK

**Keywords:** endometrial cancer, steroid receptors, aromatase inhibitors, progression free survival, overall survival

## Abstract

Objective: Although endometrial cancer (EC) is a hormone dependent neoplasm, there are no recommendations for the determination of steroid hormone receptors in the tumor tissue and no hormone therapy has ever been assessed in the adjuvant setting. The purpose of this study was to explore the effect of adjuvant aromatase inhibitors (AIs) on progression-free survival (PFS) and overall survival (OS) in patients with early stage and steroid receptors-positive EC. Methods: We retrospectively analyzed clinical and pathological factors in 73 patients with high-risk (49.3%) or low-risk (50.7%) stage I (*n* = 71) or II (*n* = 2) endometrial cancer who received by their preference after counseling either no treatment (reference group) or AI. Prognostic factors were well balanced between groups. Expression of estrogen receptor (ER), progesterone receptor (PgR), and Ki-67 index was correlated with clinical outcomes. Results: Univariate and multivariate Cox proportional regression analyses, adjusted for age, grade, stage, depth of myometrial invasion, lymphovascular space invasion, BMI, ER, PgR and Ki-67 labeling index levels, showed that PFS and OS had a trend to be longer in patients receiving AI than in the reference group HR= 0.23 (95% CI; 0.04–1.27) for PFS and HR= 0.11 (95% CI; 0.01–1.36) for OS. Conclusion: Compared with no treatment, AI exhibited a trend toward a benefit on PFS and OS in patients with early stage hormone receptor-positive EC. Given the exploratory nature of our study, randomized clinical trials for ER/PgR positive EC patients are warranted to assess the clinical benefit of AI and the potential predictive role of steroid receptors and Ki-67.

## 1. Introduction

Endometrial cancer (EC) is the most common cancer of the female reproductive organs affecting mainly postmenopausal women with an average age at diagnosis of 60 years. The American Cancer Society estimates about 58,000 new cases and more than 10,000 deaths in 2018 in the US [1]. EC represents over 90% of uterine cancer and develops along two different pathways with distinct molecular alterations, histologic and clinical types [2]. The majority (~80%) of ECs have endometrioid differentiation, are associated with an excess of estrogens related to obesity and insulin resistance (type I) and are usually detected at an early stage [3]. Conversely, the remaining ~20% ECs of unknown etiology (type II) are diagnosed at a more advanced stage, tend to be more aggressive and their risk factors are less well identified but may include women with inherited microsatellite instability [2]. Estrogen (ER) and progesterone (PgR) receptor expression is linked to the grade of histology differentiation in EC with 70%, 55% and 41% of grade 1, 2 and 3, respectively [4]. The recent analysis of the Cancer Genome Atlas Project (TCGA) confirmed an increased expression of ER and PgR in association with the grade of histological differentiation in EC [4]. The incidence of EC has been found to directly correlate with the increase in body mass index (BMI) which is an independent risk factor for this disease [2]. The relative risk of cancer-specific mortality for obese women (BMI = 30–34.9 kg/m^2^) is more than doubled compared with women with a normal range BMI (RR = 2.53 (95% CI; 2.02–3.18)) and is much greater for women with BMI > 40.0 kg/m^2^ (RR = 6.25 (95% CI; 3.75–10.42)) [2]. In addition, uterine endometrial cell proliferation is under the control of both estrogen and progesterone [5] and previous clinical, biological and epidemiological studies have demonstrated that the excess of exogenous/endogenous estrogens represents one of the main risk factors for EC [6]. Moreover, prospective cohort studies have shown that increased serum estrogen levels double the risk of EC incidence particularly for type I [7,8]. Aromatase, the enzyme responsible for a key step in the biosynthesis of estrogens, produces the majority of circulating estrogen in postmenopausal women [9]. Studies have demonstrated how high levels of aromatase are expressed in EC with respect to normal endometrium leading to the hypothesis that the enzyme functions through a paracrine mechanism [10,11]. Selective estrogen receptor modulators (SERMs) or aromatase inhibitors (AIs) are being used in the palliative treatment of advanced EC, but their effect as adjuvant treatment is unknown [12]. Recently, the National Comprehensive Cancer Network (NCCN) has updated the guidelines for EC management including the use of hormone therapy for advanced low-grade endometrioid histology, preferably in patients with small tumor volume or an indolent growth pace, although recommendations are category 2A because of the lack of definitive trials [13]. This retrospective cohort study aimed to probe the effect of AI in the adjuvant setting of EC and to explore the prognostic/predictive significance of ER/PgR expression and Ki-67 levels.

## 2. Results

### 2.1. Study Population

In total, 73 patients met our inclusion criteria. The overall median age was 74.7 years (SD + 10.4). The median expression levels of steroid receptors were 80% (range, 20–40) for ER and 70% (range, 30–90) for PgR and 40% (range, 30–65) for Ki-67. A depth ≥ 50% of myometrial invasion was present in 45.2% of patients while lymphovascular space invasion was present in 5.5%, both prognostic factors for EC were well-balanced in the AIs and no-treatment group. Overall, 50.7% were low risk and 49.3% were at high risk according the ESMO guidelines [14,15]. The 38.3% of the cohort population had healthy weight, 28.3% overweight and 33.3% were obese. Adjuvant treatment included AIs (exemestane or letrozole, 71% and 29%, respectively). The choice of the AIs was based on patient preference after medical counseling on the toxicity profile of each compound, and was offered to all the patients [16,17]. The main patient characteristics and prognostic factors were evenly distributed between groups and are summarized in Table 1.

### 2.2. Efficacy

**Figure 1:**
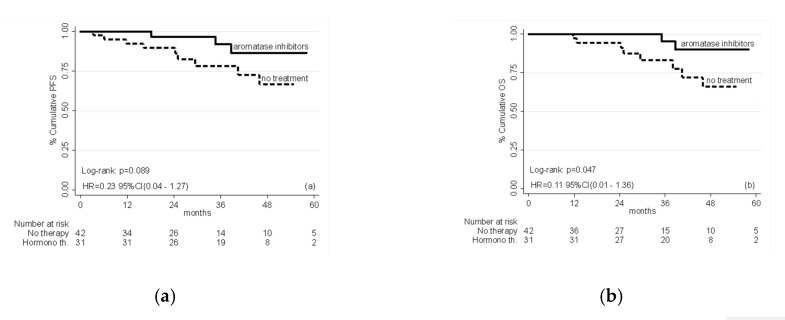
(**a**) Progression-free survival (left panel) and (**b**) overall survival (right panel) in patients treated with adjuvant hormone therapy or no treatment; HR adjusted for age, BMI, grade, depth of myometrial invasion, grading, lymphovascular space invasion, ER and PgR expression levels and Ki-67 labeling index.

At the time of the present analysis, 62 out of 73 (86.3%) patients were alive. Kaplan–Meier survival curves for median progression-free survival (PFS) and overall survival (OS) are shown in Figure 1. The choice for hormone therapy was offered to all the patients and the type of treatment was decided by patient preference but it is interesting to note that the treatment arms with AIs and no-therapy were well balanced in terms of prognostic factors. Multivariate analysis, adjusted for age, BMI, grade, depth of myometrial invasion, lymphovascular space invasion, ER and PgR expression levels and Ki-67 labeling index, showed a ~80% relative reduction in the risk of progression in patients treated with AIs over the untreated group in terms of PFS (HR = 0.23 (95% CI; 0.04–1.27); *p* = 0.089) (Figure 1a). Likewise, women in the AIs group exhibited an 89% relative reduction in the risk of death compared with the untreated group (HR = 0.11 (95% CI; 0.01–1.36); *p* = 0.047) (Figure 1b).

In addition, patients with age > 70 years and high Ki-67 levels were associated with a higher risk of shorter PFS and OS (Table 2 and Table 3). Intriguingly, while ER expression was associated with longer PFS and OS, PgR expression had a direct association with shorter PFS and OS (Table 2 and Table 3). No severe adverse events have been reported and the most common toxicity was a mild arthralgia which has never led to treatment disruption.

## 3. Discussion

Although EC is a hormone-dependent neoplasm, to date the standard treatment of early stage EC includes mainly surgery and/or radiotherapy and/or chemotherapy. Hormone therapy is recommended by the NCCN only in advanced EC patients, with low grade, low volume and slow growth rate tumors. It is also considered for early stage patients who are not suitable for primary surgery, in selected cases. [13]. Hormone therapy could represent a tolerable and effective therapeutic option in the adjuvant setting but the lack of RCTs for evaluating its efficacy does not allow clarifying the clinical benefit in terms of PFS and OS. It is well known that estrogen is the most significant risk factor for type I EC and the results of genome analysis confirmed the association of high ER/PgR expression levels with endometrioid histology suggesting a prognostic role of steroid receptors. Thus, we performed a retrospective study to explore the effect of AIs in terms of PFS and OS versus no therapy in ER/PgR positive early stage EC patients and to determine the prognostic and predictive role of ER/PgR to adjuvant endocrine therapy. Treatment with letrozole or exemestane was offered to all the patients based on clinical characteristics and patients opted for either AIs or no treatment based on their preference after accurate counseling. The two groups were well balanced for prognostic factors and our prior experience with a similar patient-decision approach in women with breast ductal carcinoma in situ [18] and in breast intraepithelial neoplasia using low dose tamoxifen indicates that the results are in line with those obtained in a subsequent RCT [19]. In this context, our results suggest a potential clinical benefit in AIs arm compared to untreated patients with regard to PFS (HR = 0.23 (95% CI; 0.04–1.27)) and OS (HR = 0.11 (95% CI; 0.01–1.36)) and are worth exploring in a randomized trial.

Interestingly, no clinical trial of AIs in an adjuvant setting of early stage EC has ever been completed or is underway [20]. The reason for this gap in this unmet medical area is unclear, but may in part be due to the lack of commercial interest of AIs, which are all out of patent, thus making a non-profit clinical trial difficult to conduct for financial constraints. Tamoxifen could also be an alternative to AIs given its favorable safety profile, especially at a lower dose where efficacy is comparable to the standard dose in early breast cancer [19]. Importantly, a progestin such as medroxyprogesterone acetate was assessed in a randomized trial in the early 1990s [21]. The results were negative in terms of PFS. Our findings seem to suggest that PgR expression is associated with worse prognosis and this might explain why giving a progestin may not be effective. Our findings also advise that Ki-67 labeling index may be a prognostic factor in EC, in line with previous data [14,22,23,24].

Study limitations are the non-randomized nature of the study and the small sample size, which only provides exploratory evidence. However, despite the retrospective nature of the study, there are no significant differences in terms of prognostic factors and/or possible confounders, i.e., the two treatment arms are comparable for each other factor different from the treatment (*p* > 0.3). The Multiple Cox model, adjusted for possible confounders, confirms the findings of the univariate analysis presented in Table 1 and in the survival curves (Figure 1). In conclusion, our results suggest that AI is a useful treatment modality to prolong PFS and possibly OS in the adjuvant treatment of patients with steroid receptors-positive EC. Randomized trials assessing the efficacy of AIs are warranted to confirm our findings.

## 4. Materials and Methods

### 4.1. Patients

This is a retrospective survival study conducted between January 2011 and December 2017 on 73 women operated for EC at Galliera Hospital. The study was approved by the Regional Ethical Committee (code 214-2018, 25 March 2019). Demographic, clinical, pathologic, and follow-up data were obtained from patients’ medical records. The study only included patients with ER/PgR positive tumors, criterion for prescription of hormone therapy and the median follow-up was 39 months. The choice for hormone therapy was offered to all the patients and the decision to undergo an aromatase inhibitor or no treatment in women with stage I EC was based on patient preference after careful medical counseling by a single medical oncologist (ADC). Patients were treated with Exemestane 25 mg once daily or Letrozole 2.5 mg once daily for 2 years. The inclusion criteria were: (1) histologically diagnosed stage I or II EC and positive hormone receptor expression; (2) patients operated for EC in the period 2011–2017 at the Galliera Hospital; (3) age > 18 years; (4) no contraindication to AIs use, including any prior cancer, prior cardiovascular disease, osteoporosis, grade 2 or higher biochemical alterations, prior use of selective estrogen receptor modulators or aromatase inhibitors, or mental disorders. Patient response to therapy and survival rates were correlated with demographic and clinic-pathologic variables to evaluate factors associated with the response to AIs, including a high risk of relapse according to the ESMO guideline which comprises FIGO stage and lymphovascular space invasion while the kind of surgery, the nodal status and the number of positive nodes are not included for the determination of the risk of recurrence [15,25].

### 4.2. Tissue Sampling, Histopathological Analysis and Immunohistochemistry (IHC)

Tumor sampling was performed immediately after hysterectomy. For histopathologic examination, 2 μm-thick FFPE sections were stained with the conventional hematoxylin and eosin stain. Two pathologists confirmed the diagnosis of endometrial carcinoma. IHC was performed on 2 µm sections with an automated IHC staining system (Ventana BenchMark ULTRA, Ventana Medical Systems, Italy).

All cases were stained IHC with Estrogen (clone SP1, 1/100, Ventana), Progesterone (clone 1E2, 1/100, Ventana) and Ki67/MIB1 (clone 30-9, 1/100, Vantana). Glands and endometrial stroma constituted the internal positive control for IHC procedure. Two independent observers scored the slides.

### 4.3. Statistical Analysis

PFS refers to the period from the start of treatment administration to disease progression or death by any cause; OS is defined as the period from the start of treatment administration to death by any cause, or the last follow up. The final data cutoff date was 31 December 2018. The sample was explored with descriptive statistics in order to highlight the difference between arms of treatment. Common indices like mean (SD) and median (IQR) were adopted to analyze continuous variables, *t*-Test or Kruskal–Wallis ANOVA were used to test the equality of mean or median between the two groups of treatment. Categorical variables were described in terms of frequencies and the comparison between the two groups was done with the Fisher’s exact test. PFS and OS between the two comparisons of interest, i.e., AIs versus no treatment, were assessed with Kaplan–Meier estimator and at an explorative level the difference between two survival curves was tested by using the log-rank test. An adjusted Cox PH model was adopted to quantify and test the difference of hazard between groups for both PFS and OS. Threshold of significance was set alpha = 5%, two-tailed. All the analyses were done with software STATA (Stata Corp. 2015. Release 14.2. College Station, TX: Stata Corp LP).

### 4.4. Assessment of Therapeutic Option and Patients Counselling

Patients were stratified into risk classes in accordance with the recent ESMO guidelines [15,25]; on the basis of biological characteristics of tumors, patients have been therefore assigned to three classes of risk (low, intermediate and high) of disease recurrence. Therapies were generally decided by clinicians according to practical clinical guidelines, which just take into account the risk of recurrence.

However, it has not always been possible to administer the therapies indicated according to guidelines because of the general health status, age and preferences of patients. So, in many cases, hormonal therapy was proposed as an alternative opportunity for those patients who should have undergone chemotherapy or radiotherapy but who lacked these treatments for the reasons explained above.

## Figures and Tables

**Table 1:** Patients’ characteristics.

Variable		Overall *N* = 73 (100%)	Hormone Therapy *N* = 31 (42.5%)	No Treatment *N* = 42 (57.5%)	*p*-Value
**Age, mean (SD)**		74.6 (10.4)	73.4 (9.7)	75.5 (10.9)	0.396 ^2^
**Age**, N (%)	<70 yrs	25 (34.3)	11 (35.5)	14 (33.3)	1.000 ^1^
	>70 yrs	48 (65.8)	20 (64.5)	28 (66.7)	
**BMI**, N (%)	healthy weight	22 (30.1%)	9 (29.0%)	13 (30.9%)	0.912 ^1^
	over weight	31 (42.5%)	14 (45.2%)	17 (40.5%)	
	obesity	20 (27.4%)	8 (25.8%)	12 (28.6%)	
**Adjuvant treatment:** **Exemestane** **Letrozole**		22 (71%) 9 (29%)	22 (71%) 9 (29%)	0 (0%) 0 (0%)	-
**Depth of myometrial invasion**, N (%)	<50%	40 (54.8)	18 (58.1)	22 (52.4)	0.644 ^1^
	>50%	33 (45.2)	13 (41.9)	20 (47.6)	
**Stage**, N (%)	I	56 (82.4)	26 (86.7)	30 (79.0)	0.720 ^1^
	II	7 (10.3)	2 (6.7)	5 (13.2)	
	III	5 (7.4)	2 (6.7)	3 (7.9)	
**Grade**, N (%)	<G2	36 (50.7)	14 (46.7)	22 (53.7)	0.617 ^1^
	>G3	35 (49.3)	16 (53.3)	19 (46.3)	
**Lymphovascular space invasion**, *N* (%)	No	69 (94.5)	29 (93.5)	40 (95.2)	1.000 ^1^
	Yes	4 (5.5)	2 (6.5)	2 (4.8)	
**ER**, median (IQR)		80 (20–90)	80 (40–90)	80 (20–90)	0.499 ^3^
**PgR**, median (IQR)		70 (30–90)	80 (50–90)	70 (10–90)	0.358 ^3^
**Ki-67**, median (IQR)		40 (30–65)	40 (20–60)	50 (30–70)	0.308 ^3^

(1) Fisher’s Exact for frequencies; (2) *t*-test for means; (3) Mann-Whitney test for equality of medians.

**Table 2:** Association between variables and progression-free survival (PFS) in multivariate Cox model.

PFS	HR	95% CI	*p*-Value
AI vs. no therapy	0.23	0.04–1.27	0.092
Age > 70	9.89	0.93–105.53	0.058
BMI 25–29 vs. BMI < 25	0.28	0.05–1.55	0.144
BMI > 30 vs. BMI < 25	0.44	0.06–3.12	0.409
Myometrium invasion (yes vs. no)	0.87	0.19–3.94	0.854
Grade > G2	1.02	0.23–4.53	0.974
Lymphovascular space invasion (yes vs. no)	3.33	0.32–34.49	0.314
ER	0.97	0.93–1.01	0.178
PgR	1.03	0.98–1.09	0.209
Ki67	1.04	1.01–1.09	0.026

**Table 3:** Association between variables and overall survival (OS) in multivariate Cox model.

OS	HR	95% CI	*p*-Value
AI vs. no therapy	0.11	0.01–1.36	0.085
Age > 70	Na *	Na *	Na *
BMI 25–29 vs. BMI < 25	0.18	0.03–0.97	0.046
BMI > 30 vs. BMI < 25	0.21	0.01–3.03	0.253
Myometrium invasion (yes vs. no)	1.04	0.22–4.92	0.962
Grade > G2	0.80	0.14–4.71	0.803
Lymphovascular space invasion (yes vs. no)	2.66	0.21–33.14	0.446
ER	0.96	0.90–1.02	0.199
PgR	1.05	0.97–1.13	0.204
Ki67	1.06	1.01–1.11	0.027

(*) not estimable due to collinearity with the outcome.
